# Advancing Neurolinguistics in Russia: Experience and Implications of Building Experimental Research and Evidence-Based Practices

**DOI:** 10.3389/fpsyg.2021.702038

**Published:** 2021-09-03

**Authors:** Maria V. Ivanova, Svetlana Malyutina, Olga Dragoy

**Affiliations:** ^1^Aphasia Recovery Lab, Department of Psychology, University of California, Berkley, Berkley, CA, United States; ^2^Center for Language and Brain, HSE University, Moscow, Russia; ^3^Department of Experimental Study of Speech, Institute of Linguistics, Russian Academy of Sciences, Moscow, Russia

**Keywords:** neuroscience, neurolinguistics, aphasia, education, public outreach, program development

## Abstract

Russia has rich theoretical and behavioral research traditions in neurolinguistics and neuropsychology, but at the beginning of the twenty-first century contemporary experimental research in these disciplines remained limited, leading to proliferation of non-evidence-based approaches in education, healthcare, and public beliefs. An academic response to this was the establishment of the Center for Language and Brain at the HSE University, Moscow, which focused on experimental psycho- and neurolinguistic research and related evidence-based practices. The Center has grown from a small group of young researchers to a large interdisciplinary unit that conducts cutting-edge research utilizing multi-site settings and novel structural and functional neuroimaging methods. The overarching aim of the Center's research is to promote scientifically grounded treatment of the language-brain relationship in the educational, clinical, and industry settings. Specifically, translational research at the Center is contributing to the advancement of clinical practice in Russia: from providing the first standardized aphasia language test to implementing protocols for intraoperative language mapping in neurosurgery departments across the country. Within research projects, a new generation of scientists is successfully being fostered, while a broader student audience is reached via courses taught by staff of the Center to students of different majors. Notable examples of public outreach programs at the Center are the Annual Summer Neurolinguistics School attracting hundreds of attendees from different countries each year, and community projects focused on raising awareness about aphasia. Together, these efforts aim to increase scientific knowledge in a multi-professional audience. In this paper, we will share our joint experiences in establishing, building, and promoting a neurolinguistics research center in Russia and the impact that this work has had on the broader public. We will delineate specific milestones of this journey and focus on the main pillars that have contributed to our progress: research, clinical work, teaching, and public outreach programs. We hope that this critical appraisal of our experiences can serve simultaneously as an inspiration and a practical guide for other groups developing research, clinical, and educational programs in different neuroscientific disciplines across the globe and aiming to improve the quality of the neuroscientific information available to the public.

## Introduction

Russia has rich theoretical and behavioral research traditions in linguistics, starting in the late nineteenth and early twentieth century with the works of, among others, Ivan Baudouin de Courtenay and Lev Shcherba, and continuing with pioneering studies in structural linguistics by Roman Jakobson and more contemporary works in psycholinguistics by Revekka Frumkina, Alexey Leontiev, Stella Tseitlin, Tatiana Chernigovskaya and many others (Berezin, 1984; Alpatov, 2005). Similarly, the Russian neuropsychology school formed by Alexander Luria in the middle of the twentieth century has been very prolific in clinical research (Luria, 1980) and influential within and outside of Russia (Tupper, 1999). Despite this heritage, research in psycho- and neurolinguistics in the beginning of the twenty-first century remained fragmented and often only qualitative (for a critical review, see Fedorova, 2020). Only scattered studies have employed sound empirical methods for behavioral psycholinguistic research (e.g., Fedorova, 2009) or for further elaborating Luria's theory of higher cortical functions (e.g., Homskaya and Moskvin, 2000; Akhutina, 2002). The lack of a systematic scientific approach and a strong experimental school that would include neuroscience methods inevitably led to proliferation of non-evidence-based approaches in education, healthcare, and public beliefs.

**Figure 1:**
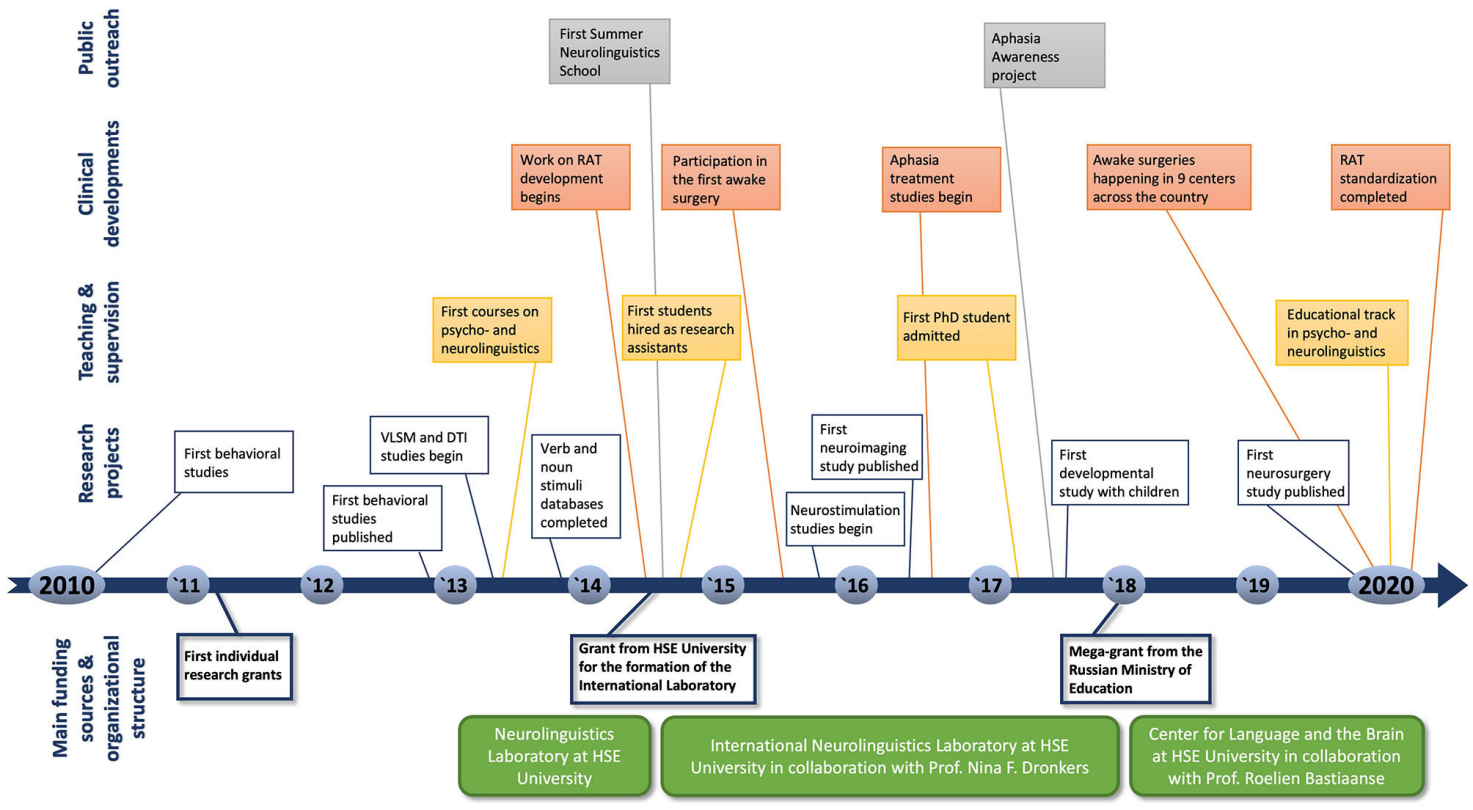
Timeline representing the advancements and contributions made by our team to the field of neurolinguistics in Russia in 2010–2020, highlighting the main milestones and accomplishments in research, clinical work, teaching and supervision, and public outreach programs.

In 2009–2010, a small initiative group of young researchers with backgrounds in linguistics, speech-language pathology, and neuropsychology began a series of behavioral studies into language and memory, followed by application for independent funding to Russian research agencies. These investigations served as the foundation for the Neurolinguistics Laboratory, co-founded by Dr. Olga Dragoy and Dr. Maria Ivanova 3 years later at the HSE University in the framework of the HSE Basic Research Program. In 2014, with additional funding from the HSE University as a part of the Russian Academic Excellence Project 5-100, it became the International Neurolinguistics Laboratory headed by Dr. Olga Dragoy and co-headed by Dr. Maria Ivanova with guidance from a senior scientist and a prominent figure in the field, Prof. Nina F. Dronkers (University of California Berkley, U.S.), who took on the role of the scientific advisor for the laboratory during its first 3 years. Incrementally, through collaborations with numerous leading international scientists, the diverse empirical behavioral inquiries broadened to include many cutting-edge neuroimaging methods: from lesion-symptom mapping approaches to electrocorticography. For the next stage of development, the laboratory was able to receive the prestigious mega-grant from the Russian Government in 2017. In 2018, with that funding under the leadership of Dr. Olga Dragoy and co-headed by Dr. Svetlana Malyutina with the distinguished neurolinguist Prof. Roelien Bastiaanse (University of Groningen, the Netherlands) as the scientific advisor, the Center for Language and Brain was founded on the basis of the Neurolinguistics Laboratory. In addition to these larger sources of funding to support the main research program, the team has obtained numerous smaller grants from public agencies (Russian Foundation for Humanities, Russian Foundation for Basic Research, Russian Science Foundation) for individual and exploratory projects. Today, a wide range of research projects on cognitive and neural mechanisms of language and related cognitive functions in diverse typical and atypical populations are conducted at the Center. The overarching aim of the Center's continuously expanding research program is to promote scientifically grounded treatment of the language-brain relationship in the educational, clinical, and industry settings. The main milestones and highlights of this decade-long (and still continuing) journey are presented on a timeline in Figure 1.

In this paper, we will share our joint experiences in establishing, building, and promoting a neurolinguistics research center in Russia and the impact of this work on the broader community. We will delineate specific milestones of this journey and focus on the four main pillars that have contributed to our progress: research, clinical work, teaching, and public outreach programs. We will discuss distinct actions that have been particularly effective and pitfalls that we encountered along the way. We hope that this appraisal of our experiences can serve simultaneously as an inspiration and a practical guide for other groups developing research, clinical, and educational programs in different neuroscientific disciplines across the globe and aiming to improve the quality of the neuroscientific information available to the public.

## Research Advancements

Research was the starting point in the development of the Neurolinguistics Laboratory and to this day remains the main driving force of the Center's growth, uniting other areas of service and activity, such as clinical, teaching, and public outreach work.

A decade ago, the research began with behavioral psycholinguistic studies in healthy participants and individuals with post-stroke aphasia. The authors of this paper along with several other junior researchers and students at first used behavioral methods alongside eye tracking to explore the cognitive and linguistic mechanisms supporting sentence comprehension. A collaboration with the Center for Speech Pathology and Neurorehabilitation in Moscow (which would later become the Center's prime clinical partner on numerous projects) enabled us to access the population of patients with neurogenic communication disorders after stroke and to use the Center's eye tracking system for experimental research. This series of behavioral experiments laid the groundwork for programmatic research on linguistic and cognitive mechanisms of typical and atypical language processing. The use of eye movement measures in addition to offline behavioral methods allowed to keep up with modern trends in psycholinguistics, where particular emphasis is placed on understanding online language processing. These investigations offered insights into the contribution of different memory and attention processes in healthy participants and individuals with neurogenic language disorders (Laurinavichyute et al., 2014; Ivanova et al., 2015). The first studies and subsequent publications were critical in establishing the group's scientific competencies and helped to obtain subsequent funding. Additionally, these first investigations solidified the first clinical collaborations that would be vital for further development.

At the same time, our team realized that in order to establish a strong research program in psycho and neurolinguistics, a sound foundation was needed: a database of experimental stimuli with established and validated properties. Compared to English and other European languages, Russian lacked publicly available databases of word properties beyond lexical frequency. To fulfill this gap, work on verb and noun databases began. Our group normed an extensive list of verbs and nouns by collecting data on relevant psycholinguistic word properties (age of acquisition, imageability, and image agreement) along with parameters of corresponding visual stimuli (name agreement, action/object familiarity, and subjective image complexity) through online questionnaires (Akinina et al., 2014, 2015). This allowed to create a psycholinguistic database of stimuli that served as a solid foundation for future research and clinical work. For instance, the assessment instruments developed by our group (such as the Russian Aphasia Test—see next section for more details) are largely based on stimuli from these databases. Many of our subsequent experiments rely on these stimuli as well (e.g., Yurchenko et al., 2017; Soloukhina and Ivanova, 2018; Malyutina and Zelenkova, 2020). In other words, without this groundwork of creating a database of stimuli, the next set of projects would not be possible. The databases have been made publicly available (http://en.stim-database.ru/) and are also being used by other research groups studying the Russian language. Moreover, a database in the Tatar language, the second most common language in Russia, has been created (http://stim-database.ru/ru-tatar/). Together, these efforts laid the foundation for quantitative psycho- and neurolinguistic research in the Russian-speaking population.

Our first steps in experimental psycholinguistic research were inevitably related to the specific properties of the language we dealt with—Russian. Most contemporary psycho- and neurolinguistic models rely on English and at best take into account some other Indo-European Germanic or Roman languages (e.g., Dutch, German, Spanish). In contrast to them, Russian as a representative of Slavic languages, has by far a more developed morphosyntactic system: three genders, six cases, at least three traditionally distinguished declension types and two conjugation types, lexical-grammatical aspect, agreement in case, gender and number, complex system of morphonological alternations, and free word order. On one hand, these differences made it challenging to directly adopt Anglocentric models to a wide range of linguistic phenomena. But on the other hand, these disparities afforded numerous opportunities for more careful testing and further exploration of existing psycholinguistic models. One such example is our eye-tracking experiment that allowed to reinterpret filler reactivation at the trace position in wh-questions, due to the use of specific constructions existing in Russian, but not in English (Sekerina et al., 2019). In another study, free word order and case marking in Russian allowed us to comprehensively test the impact of isomorphism as a linear agreement between the order of sentence constituents and the temporal sequence of events on sentence processing (Dragoy et al., 2016b; Chrabaszcz et al., in press). Thus, overall, specific features of experimentally understudied languages enable to refine existing linguistic models and afford new generalizations about language processing. The integration of such a language into the global research agenda might not be easy in the beginning but is ultimately rewarding.

After successfully completing several behavioral and eye tracking projects and having developed a stimuli database, we felt ready to start tackling the least addressed area in Russian experimental linguistic research—the neural mechanisms underpinning cognitive and linguistic processes. This required becoming proficient at using neuroimaging methods and collaborating with institutions that had this research infrastructure. Here, partnerships with clinical sites established while conducting our first behavioral and eye tracking experiments enabled us to access MRI scanners and EEG systems at these facilities. We began mastering fMRI and ERP methods by collaborating with mentors in Germany (Prof. Ernst Pöppel and Dr. Evgeny Gutyrchik, Ludwig Maximilian University of Munich) and the Netherlands (Prof. Laurie Stowe and Prof. Roelien Bastiaanse, University of Groningen) and successfully completed studies in healthy individuals (Dragoy et al., 2012; Yurchenko et al., 2013; Malyutina et al., 2016). We then attempted to apply functional neuroimaging to investigate language processing in patients after stroke and invested effort into starting several projects. However, our expertise at the moment was not sufficient to counter various methodological and conceptual issues inherent to application of functional neuroimaging in the lesioned brain (Specht, 2020), so these projects were discontinued.

Given the historical legacy of Luria's neuropsychology and his lesion approach to understanding the neural substrate of cognitive functions (Luria, 1980), as well as our extensive work with patients with focal lesions following stroke, we were very much interested in pursuing contemporary lesion methods. So, next, having learned the basics of MRI data collection and processing, we started learning modern lesion analysis techniques under the guidance of Prof. Nina F. Dronkers, one of the pioneers of the voxel-based lesion symptom mapping method (Bates et al., 2003). This method allows to evaluate contribution of individual voxels in the brain to the function of interest through statistical modeling. Using this method, our group determined neural regions critical for working memory (Ivanova et al., 2018) and verb naming (Akinina et al., 2019). Currently, we are using the method to explore the neural substrates of different aphasia types, bringing Luria's classification of aphasias into the contemporary neuroscience context (Luria, 1980). Another technique that we have adopted involves diffusion-weighted imaging and tractography analyses (e.g., Ivanova et al., 2016; Zyryanov et al., 2020). These methods allow to investigate the integrity of white-matter fiber pathways in the brain and determine their functional specialization. At this time, more advanced methods such as electrocorticography are also being used, along with further development and elaboration of behavioral and eye tracking studies.

In research development, the key to success has been integration of research with clinical work and incrementality in building a research program. Here, we would really like to emphasize the need to start with short manageable projects. Studies where results can be obtained on a realistic 1–2 years' time scale will serve as a great starting point and foundation for larger projects. Along the same lines, it is advantageous to start with more simple and straightforward methods that are easier to implement compared to more sophisticated neuroimaging techniques. Importantly, it is recommended to explore a method in-depth and complete a single project with it to understand the potential hidden caveats, before using it more widely. In this regard, we clearly made a planning mistake by initially investing a lot of resources into functional neuroimaging studies of language in patients with focal lesions, while the applicability of this method to the stroke population proved to be too tortuous and confounded for our level of expertise then, leading our group to abandon several functional neuroimaging projects without coming to specific results. In hindsight, it would have been more effective to conduct a single functional neuroimaging study and fully complete it, prior to starting other inquiries using the same method.

On the contrary, a prominent example of successful incrementality in research has been our line of lesion studies: it began with the investigation of a specific syndrome (semantic aphasia, Dragoy et al., 2017), followed by larger group studies and more advanced methods (voxel-based lesion symptom mapping, Ivanova et al., 2018; Akinina et al., 2019), with current efforts focused on creating a large lesion database to investigate the neural substrate of different aphasia types. Specifically with regard to lesion symptom mapping, our group has been able to effectively conduct several studies on the same cohort, again something that is highly desirable given the resources involved in carrying out any kind of large group neuroimaging studies with a clinical population. Generally, in the initial stages of development, we would like to warn against getting involved in large-scale projects that are time-consuming, require experience managing a large team and data from multiple sites, and do not yield tractable outcomes in terms of research findings and practical recommendations, as in the beginning it is vital to establish oneself as a group that can achieve stated results.

Further, initial collaborations with internationally renowned experts on joint projects provided the much-needed mentorship and guidance on mastering new skills, while close alliances with clinical sites afforded access to the infrastructure needed for this work (e.g., MRI scanners, EEG systems) and clinical populations. In general, we believe that it is beneficial to have a fluid research agenda in the beginning of establishing a research center. Being open to new avenues of research, new collaborations and new methods will lead to unexpected opportunities, higher productivity and multi-faceted outcomes.

## Clinical Developments

Given the interdisciplinary nature of the field of neurolinguistics and the current trends in clinical neurolinguistics in the West, from the beginning we realized that through our research we needed to address practical needs of clinicians working with varied groups of patients with language disorders. In short, we wanted to make a meaningful contribution to improved clinical practice in Russia. We saw two main gaps in clinical work that we felt could be effectively addressed by our group: development of contemporary assessment tools and advancement of novel treatment approaches.

While Russian is one of the ten most commonly spoken languages in the world, there is a clear lack of standardized language assessment tests in Russian (Ivanova and Hallowell, 2013). Historically, a qualitative approach to assessment grounded in Luria's neuropsychological theory has dominated the clinical field in Russia (Luria, 1980; Akhutina, 2016). While this descriptive, qualitative approach is valuable in understanding the mechanisms of cognitive impairments and their neural substrate, it is not readily quantifiable, lacks generalization, and is highly dependent on the expertise of the clinician doing the assessment. As such, lack of standardized measures makes it impossible to provide description of patients in research studies, systematically explore neural mechanisms of language deficits and compare findings cross-linguistically. In terms of clinical work, it makes it challenging to compare patients and protocols across different hospitals and evaluate efficacy of treatments. Thus, when implemented exclusively on its own, the traditional neuropsychological qualitative approach impedes evidence-based practice and research that is contingent on having valid and reliable instruments to quantitatively measure cognitive and language impairments. With a team of linguists, speech-language pathologists, neuropsychologists, and computer scientists, we decided to proactively address this methodological gap.

So, one of the first and most prominent clinical research projects conducted at the International Neurolinguistics Laboratory was the creation, development, standardization and then clinical implementation of a novel comprehensive aphasia test. The aim was to develop a quantitative language battery that was both comprehensive and yet compact to be administered in a clinically feasible time. In 2014, using previously accumulated knowledge on test development (Ivanova and Hallowell, 2013) and clinical expertise, the Russian Aphasia Test (RAT; Ivanova et al., 2021) was designed. The test is meant to provide a multidimensional characterization of impaired and spared aspects of language functioning. The RAT evaluates the critical linguistic levels of processing (phonological, lexical-semantic, syntactic, and discourse) in three different domains: auditory comprehension, repetition, and oral production. During subtest design and stimuli development we took into account various (psycho)linguistic factors known to impact language processing, as well as distinct properties of the Russian language. For instance, consonant distinctive features specific to Russian were manipulated in the nonword discrimination subtest. In the single word comprehension and naming subtests item selection was based on the stimuli database developed earlier by our group (see previous section for more information, Akinina et al., 2014, 2015, http://en.stim-database.ru/) allowing us to account for a number of relevant psycholinguistic parameters (imageability, age of acquisition, name agreement, image agreement, object/action familiarity, visual complexity) in addition to the standard measure of lexical frequency. The sentence comprehension and production subtests took advantage of the flexible word order in Russian to investigate processing of canonical versus noncanonical sentences (see Ivanova et al., 2021 for more details). The test's initial piloting, subsequent extensive normative data collection and standardization involved hundreds of participants and took 5 years (2014–2019). Also, for the final version of the test, our group developed a tabled-based version of the RAT, which further enhanced uniformity of administration, simplified and standardized scoring procedures, facilitating data collection in clinical and research settings. This titanic work has just recently been fully completed (Ivanova et al., 2021).

However, the test's development was not without complications and caveats along the way. This project was overly ambitious at the time it was conceived in 2014, as back then our group had limited experience with test development and organizing such a large-scale project. This led to many predictable blunders along the way: difficulty managing data collected from a large team of students and clinicians; alterations made to the test materials and its structure after standardization has started, requiring repeated data collection; changing technical platforms during the standardization phase, leading to painstakingly manual data aggregation and recoding; and, finally, altering scoring guidelines several times during data analysis requiring complete rescoring of all protocols. In hindsight, we could have implemented this project much more efficiently and with less resources had we started with test adaptation instead of development and focused on select domains and shorter tests, postponing the bigger project for a few years. Today, following tumultuous but eventually successful navigation of logistical and procedural hurdles along the way, the test is now being widely distributed in Russia and is actively used in several large stroke rehabilitation Centers. Additionally, a Tatar language version of the test has been created and is currently in the final stages of standardization (as mentioned previously, it is the second most common language in modern Russia). So, apart from these organizational shortcomings, in the end this project is a poster child of an interdisciplinary project where scientific knowledge, clinical expertise and technological advances were successfully combined to fulfill specific practical needs and enhance clinical practice standards.

From the RAT project, several other important test development initiatives have emerged. Similarly to a lack of standardized aphasia language tests, there was a dearth of standardized quantitative tests for evaluating child language development. This made it impossible to define quantitative norms for language development in Russian and to specify the type and severity of linguistic deficits in children with different developmental disorders in clinical practice and research studies. A test of child language development, the Russian Child Language Assessment Battery (RuCLAB; Lopukhina et al., 2019), was created in 2018 based on the tasks originally implemented in the RAT (Ivanova et al., 2021), with the subtests adapted to assess children's phonological, lexical, morphosyntactic, and discourse skills in comprehension and oral production. The test has been normed in typically developing children and clinical data has been collected in various atypical populations (children with Specific Language Disorder, epilepsy, Autism Spectrum Disorder; e.g., Arutiunian et al., in press). Researchers at the Center have also adapted several other broadly recognized assessment tools into Russian language and have validated them (e.g., Verb and Sentence Test: Akinina and Bastiaanse, 2017; Token Test: Akinina et al., 2019; Aphasia Rapid Test: Buivolova et al., 2020), further contributing to improving clinical practice standards in Russian, advancing evidence-based practice and enabling research studies to be compatible with other international projects.

Another important direction for assessment development has been intraoperative language mapping in tumor patients. Our team developed a linguistically grounded assessment protocol for intraoperative mapping with the aim of preserving language function in patients undergoing surgery for brain tumor or epileptogenic tissue resection (Dragoy et al., 2016b, 2017). Collaboration with surgical centers across the country has helped to broadly distribute this knowledge, stimulate broader use of awake surgeries for language mapping and implement the protocol in clinical practice leading to improved language outcomes following surgery. This highlights how cutting-edge linguistic knowledge can be used to enhance patient outcomes and improve quality of life. Again, this project is another great example of an interdisciplinary approach to resolving a practical problem through collaboration between experts from different fields. Also, it would not be a success without extensive consultations with internationally recognized experts in the field: Dr. Peter Mariën (Free University of Brussels, Belgium), Dr. Henry Colle and Dr. Erik Robert (Algemeen Ziekenhuis Sint-Lucas, Belgium), Dr. Hugues Duffau (Montpellier University Medical Center, France), Dr. Emmanuel Mandonnet (Lariboisière Hospital, Paris, France). Further, the project demonstrates the advantages of starting with a flexible research agenda and being open to new avenues of inquiry, as initially we did not have specific plans or expertise for this line of work, only a general interest in improving language outcomes in varied clinical populations.

The second main direction of our clinical work has been development, adaptation, and promotion of evidence-based speech-language treatment approaches. As in the case of assessments, language therapies used in clinical practice in Russia have remained varied and largely untested. Typically, they are selected based on the clinician's judgement in the absence of quantitative evidence base, so they are again highly dependent on the clinician's expertise. Our group has adapted two contemporary language therapies that were originally developed and proved effective in other languages: Verb Network Strengthening Treatment (VNeST, Edmonds, 2014) and constrained-induced language therapy (CILT, Pulvermüller et al., 2001). We have been conducting a series of studies testing the efficacy of their Russian adaptations (CILT: Ulanov et al., 2019; VNeST: Razmyslovich et al., 2021) in therapy protocols with and without concurrent non-invasive brain stimulation (transcranial direct current stimulation and transcranial magnetic stimulation). We had hoped that these studies would not only provide evidence on the efficacy of these specific treatment protocols adapted into Russian but also introduce a new standard for non-pharmaceutical treatment research in Russia.

So, unlike in the assessment direction of our clinical work, our team started with adaptations of existing therapies rather than with creating new ones, which appeared to be a reasonable choice with regard to feasibility of protocol development. Still, we have encountered several obstacles along the way of treatment studies. First and foremost, as these studies are very labour-intensive and prolonged, it has been difficult to find sufficient human resources within our team for their continuous implementation. This has been a particularly challenging issue because of the chosen experimental designs, which involve intensive language therapy (several hours daily for several weeks), multiple clinicians for group therapy, and a double-blind approach where non-invasive brain stimulation is administered by a clinician different from the one conducting the therapy. Our recommendation for new teams starting treatment studies is to carefully estimate the human resources needed for a particular therapy and experimental design in advance. A wise preliminary step before launching any treatment study would be a precise calculation of how many researchers, and for how long, are needed for participant recruitment, therapy administration, and pre- and post-treatment assessment, particularly if a double-blinded design is used, so that different team members would need to conduct the therapy and the assessment. Choosing a therapy that does not require group administration or intensive regimen and aiming for a small-sample proof-of-concept study rather than a full-scale clinical trial, in our opinion, is a wiser and a more realistic option for a first pass at treatment studies.

Another big challenge in our treatment studies has been to integrate research designs into routine clinical schedule at clinical sites where treatment studies have been conducted. For example, it has been complicated to orchestrate patient selection and pre-treatment baseline testing against a typical rehabilitation center admission timeline that leaves little time for assessment and requires starting the treatment within a very short timeframe. Having encountered this difficulty, we recommend that other new teams prior to starting the study consider whether the routine clinical schedule of the clinical site would allow sufficient time for participant recruitment and extended baseline pre-treatment assessment. If the clinical site is a rehabilitation center accepting returning patients, one solution that we have used is to select, recruit, and pre-test patients at the end of their first rehabilitation course and subsequently admit them into the treatment study during their next admission to the rehabilitation center. Another aspect to consider is whether other routine clinical practices of the clinical site (pharmaceutical treatment, other concurrent treatments such as occupational or physical therapy) could interfere with the language therapy being studied: for example, if these additional therapies/treatments are only prescribed to select patients, this could create unwanted differences between experimental and control groups in the treatment study. Thus, it is important to know in detail the routine practices of the clinical site, so that the research team can request to modify them appropriately and/or to collect relevant information about patients involved in the therapy study.

To date, our own treatment studies are still in progress, and our experience suggests that this avenue of research may not be an optimal choice for new research teams. Greater human resources, more intense involvement and long-term commitment of clinical facilities to the project seem to be the necessary prerequisites for fully-fledged treatment research. Nonetheless, while large-scale treatment studies are beyond the current ability and scope of the Center, we hope that our approach is still a step towards evidence-based clinical practice and can serve as a template that the surrounding speech-language pathology and neuropsychology communities can follow in evaluation of other therapies. Overall, for clinical projects, we would like to stress the importance of collaboration and interdisciplinarity. From the start, one should focus on developing and implementing interdisciplinary projects that combine theories and methodologies from different fields. For successful completion of clinical research projects, it is pivotal to involve researchers from different academic disciplines, specialists with different professional backgrounds along with clinicians and effectively incorporate their knowledge and skill set in design, implementation, analysis, and interpretation of findings, as we have been able to do in our most successful clinical projects to date: development of standardized tests and language mapping protocols for awake brain surgery.

## Teaching and Supervision

Our teaching activities have been multi-faceted and have gradually increased in scope and breadth over the years. First, individual courses (Experimental Linguistics, Experimental Methods in Psycho- and Neurolinguistics) were offered to students in the bachelor's and master's programs in Linguistics at the HSE University. These courses introduced the students to the basics of experimental design, contemporary psycho- and neurolinguistics theory, and provided an overview of different behavioral and neuroscience techniques. Then, another course (Psychology and Neurophysiology of Speech and Language) was offered to students in the Psychology bachelor's program at the HSE University. This course, on the contrary, assumed previous knowledge of experimental methods but introduced their specific application to the cognitive and neural bases of language processing. These courses were the first courses on psycho- and neurolinguistics at the HSE University and were enthusiastically welcomed by the students.

However, all the above-mentioned standalone courses were of introductory nature and did not include enough hours to teach any hands-on skills necessary for conducting independent research. Eventually, in 2020, the Center for Language and Brain established an educational track in Experimental Linguistics within the bachelor's program in Linguistics at the HSE University. The track expands over the last 2 years of the bachelor's program and includes three courses that provide both in-depth theoretical knowledge and hands-on experience in experimental linguistics. The first course (Psycho- and Neurolinguistics) is taught for two semesters during the first year of the track and provides the theoretical foundation in empirical research methods, neuroanatomy and neurophysiology, and an overview of modern psycho- and neurolinguistics theory. The second year includes two semester-long practically oriented courses (Practicum in Psycholinguistics, Practicum in Neurolinguistics) that address specific research topics and methods more in-depth and offer hands-on experience in experiment programming, data collection, and analysis, et cetera. To the best of our knowledge, this is one of the few undergraduate-level tracks/course series in psycho- and neurolinguistics in the world.

Besides offering individual courses and the educational track, researchers at the Center for Language and Brain have supervised “course research projects” and “summer practical training” of bachelor's and master's students at the HSE University. Both types of activities are mandatory parts of the curriculum in most Russian higher education programs. This is an important strength of the Russian higher education system, providing students with unique hands-on experience already at the undergraduate level. For “course research projects,” a student works on an individual research project over the entire academic year and, as a result, writes a research paper and typically defends a presentation. The same is expected for the mandatory bachelor's and master's theses during the last year of study. “Summer practical training” involves work on a hands-on task (for example, collecting or analyzing data) without any literature review, writing or presentation. In most programs, both types of activities are required on an annual basis and students are free to choose a topic and a supervisor. Over the years, staff of the Center for Language and Brain have supervised many “course research projects,” theses and “summer practical trainings,” typically involving students into their own real ongoing research projects. This has carried inherent risks for the supervisor in case the student fails to complete the assigned part of the project. Nevertheless, this practice has also brought amazing successes, whereby undergraduate students became the driving force of research projects and played an essential role in their successful completion (e.g., Soloukhina and Ivanova, 2018; Zyryanov et al., 2020; Savinova and Malyutina, 2021). Several of the students who completed their “course research projects” or bachelor's theses at the Center later went to study abroad to obtain their master's degree or PhD and then returned to work at the Center as research scientists.

The Center is also building a prolific PhD program, with the first student, who was admitted in 2017, successfully defending her dissertation in 2020. Six other students are currently undertaking their PhD studies under the supervision of the Center staff members, and every year several more are recruited. A recent innovation of the Russian educational system allowed publication-based PhD defenses, and our students eagerly follow this track and defend based on their already published peer-reviewed articles.

Furthermore, the Center represents the HSE University in two recognized international consortia—the European Master's in Clinical Linguistics (EMCL) and the International Doctorate for Experimental Approaches to Language And Brain (IDEALAB). In both, the Center acts as an associate partner, with a focus on aphasia, structural neuroimaging, and language mapping in awake neurosurgeries. Every year, a few EMCL students visit the Center for a 3-month internship, get integrated into the Center's research environment and write their theses co-supervised by the staff members of the Center. In 2021, our first jointly supervised IDEALAB student defended her dissertation at the University of Groningen (the Netherlands).

Several areas are still not covered by the educational activities of the Center. For example, no educational courses are currently offered to first- and second-year bachelor's students, which would have been helpful for those already starting their “course research projects” under the supervision of the researchers of the Center. Current educational activities are targeting exclusively Linguistics and Psychology students and do not involve any students of medicine-related professions, since there are no such programs at the HSE University. Most importantly, the Center has not yet established any fully independent self-contained educational programs. Still, incrementally, the Center is actively fostering a new generation of scientists, simultaneously advancing both education and research in neurolinguistics. We believe that this teaching-research cycle is absolutely central to scientific progress. Those who are actively involved in research are best enabled to teach the subject matter, bringing cutting-edge advancements to the classroom, and inspire a new generation of scientists through lively lectures, life examples, and tough questions. Young scientists, in turn, bring new energy, ideas, and skills to the research domain. Educational activities require time and effort that is inevitably taken away from research but, in the long run, we believe that this investment is essential for bringing the scientific field forward.

## Public Outreach

Since the foundation of the Neurolinguistics Laboratory, we have been actively involved in a variety of public outreach initiatives. First, current educational activity at the Center is not limited to academic courses at the University. The Center holds regular weekly meetings open to the public, entitled Neurolinguistics Thursdays, where researchers of the Center and invited guests speak about current trends in different subfields of neuroscience and discuss their research projects. Also, typically several times a year, the Center hosts workshops where a broader research community can gain practical hands-on knowledge about new research methodologies. A recent example was a workshop on voxel-based lesion symptom mapping that included hands-on sessions addressing all practical steps of using the method, including MRI data preprocessing and manual lesion delineation. These practical workshops also help foster research collaboration and establishment of clinical networks.

One of the key public educational events at the Center is the annual international Summer Neurolinguistics School that has been in session since 2014. The school is positioned both as an educational event for students entering the field and as an academic environment where more advanced researchers can discuss the latest ideas and achievements in the field. Each summer over 100 attendees from different countries come together in Moscow, Russia (or online, since 2020) to gain an in-depth understanding of a given topic presented by renowned guest lecturers. An important feature of the School is that the topic is different every year: in previous years the School has been devoted to aphasia, brain stimulation, neural oscillations, experimental studies across languages, et cetera. It has been a conscious decision to alternate topics so that staff members, students, and the local research community can broaden their horizons and expand their understanding of topics that are not within their area of expertise and that of their close colleagues. Since it aims to target a broad audience, the School has been facing many challenges. For example, since attendees are at very different levels of their education and career, the program needs to be tuned in such way that each lecture is accessible to novices while still offering new knowledge to more advanced attendees. As another example, due to a high number of attendees of different levels, the Schools so far have mostly consisted of lectures and presentations and have included only a minimal number of practical sessions. In spite of these challenges, we believe that the format that covers alternating topics and welcomes students and new researchers is of most value to the community.

The above educational events are aimed at students and professionals from related fields. Apart from them, the Center has been performing public outreach activities targeting the broader public and attempting to present research findings in a format accessible to a wide audience. These have included appearances of staff members in popular science shows, interviews to mass media, involvement with the Russian Dyslexia Association, community projects focused on raising awareness about aphasia, popular science lectures at social centers for the elderly, tours to the Center for middle and high school students, press releases about new publications on the university website. For instance, for raising aphasia awareness, the Center has designed information booklets for caregivers and “ID cards” for individuals with aphasia. They are freely available at the Center's website and paper copies have been disseminated among collaborating speech-language pathologists, so that they can distribute them to patients and caregivers. To the best of our knowledge, these are among the very few Russian-language materials about aphasia available to the public. Another example of public outreach activity of the Center are press releases about new publications. These are plain-language summaries of newly published research findings comprehensible to the broader audience. This format has been established and encouraged by the university, so press releases are placed at the university website, in both English and Russian, and offered for repost to mass media.

Unlike educational events for students and related professionals, public outreach activities of the Center have not been regular, due to the shortage of time and human resources. Nonetheless, we hope that even sporadic events or materials targeting the wider community may start word-of-mouth dissemination of current evidence-based views and research findings. Besides, the Center itself has also benefited from public outreach activities. Any outreach to a broad audience has helped with volunteer recruitment for the Center's studies. Outreach to clinicians helps to establish new collaborations and/or to learn more about current practices and needs of practicing clinicians. Outreach to high school students may inspire some of them to enroll at the university and get involved in the Center's research activities. Overall, while systematic public outreach activities currently remain beyond the Center's capacities, we have found that embracing occasional opportunities for public outreach brings mutual benefits and incrementally increases public scientific knowledge.

## The Scientific Scene in 2021

**Figure 2:**
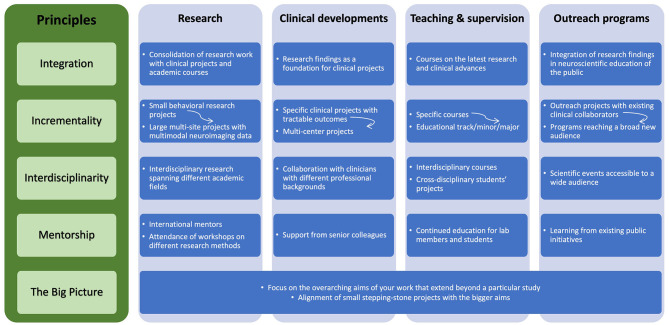
Summary of the main working principles of scientific and educational program development as they apply to different areas of activity and service (research, clinical work, teaching and supervision, and public outreach).

We have outlined above the four pillars—research, clinical work, teaching, and public outreach work—that have contributed to the advancement of neurolinguistics in Russia. This path started on pure enthusiasm with specific research projects and evolved into the largest interdisciplinary neurolinguistics research center in Russia. We would like to conclude the account of our scientific journey by summarizing working principles that we believe contributed to the success of what seemed like a very audacious endeavor a decade ago: *integration, incrementality, interdisciplinarity, mentorship*, and keeping *the Big Picture* in focus. Figure 2 provides an overview of these main working principles as they apply to different areas of activity and service (research, clinical developments, teaching and supervision, public outreach programs).

First of all, *integration* of all our lines of work has been central to our success. Intermixing and interweaving research, clinical, and academic work has been pivotal for scientific advancements and formulation of evidence-based practices. Rigorous experimental research offers a sound foundation for clinical projects and helps build an evidence base for assessment and treatment approaches. In turn, clinical interactions afford unique insights into the psychological and neural mechanisms of cognition and impart crucial motivation for research. Both research and clinical work provide irreplaceable experiences that translate into captivating teaching material. At the same time, academic work and involving students in all stages of the research process fuel research activity and enhance productivity. Together, research, clinical work, and teaching interact to support, promote and inspire each other. From the beginning one should consider carefully developing in parallel, instead of focusing on just one aspect such as research, and largely integrating these interrelated scientific activities.

In terms of *incrementality*, starting with small doable research projects seems to be the most efficient way of building comprehensive research and academic programs. Large endeavors are built on small stepping stones. This principle of incrementality also holds for funding acquisition, where starting with smaller grants and slowly building up to applying for larger grants is a more productive and feasible strategy.

While incrementally building your research program and initially being flexible in your research agenda, you should not lose track of the overarching aims of your work that extend beyond a particular study by keeping *the Big Picture* in focus. What are the big questions/issues/knowledge gaps that your group is trying to address? What change in current research, clinical, and academic practices do you hope to bring about? Aligning small stepping-stone projects with those bigger aims (such as, for example, promoting standardization and evidence-based practices in assessment of language disorders) will ensure that progress is made in the right direction leading to long-lasting impact on the field and current practices in neuroscientific disciplines.

*Interdisciplinarity* is an important aspect of contemporary research. Today, innovation and scientific advancements happen at intersection of different disciplines. Thus, from the beginning it is advantageous to include researchers and professionals with different backgrounds in your team and find collaborators from other disciplines, enabling you to successfully implement interdisciplinary projects.

Per *mentorship*, it is pivotal to find international mentors to support your journey as you begin to establish your independent programs. Again, this is particularly crucial in the first stages, as you will need advice and support on grant writing, building a professional network, and finding connections through which you can learn about new methodologies. At the same time, support and promote your students as they are the future scientists. Invest time into training them, offer interesting and motivating research experiences, endorse independent inquiries, and encourage their continuous education.

There are also several practical aspects to developing new research, clinical, and educational programs. On the funding side, it is important to ensure stability, so that staff can stay on the projects while continuing to develop professionally. Building a network of collaborating clinical sites is another vector of development that is of pivotal importance. Having access to clinical resources and different patient populations are largely key to prolific neuroscientific research. Here, from the beginning, devoting effort and time to translational and clinically motivated research is crucial, as it offers mutually beneficial interactions to clinical sites and thus promotes closer collaborations. Additionally, it is worthwhile to invest time in developing resources and procedures that will support numerous projects in the future: stimuli libraries, participant databases with behavioral and neuroimaging data, robust pipelines, digitization of data collection, script documentation for automatic data processing. While originally implementing some of these practices might be time consuming and seem almost inefficient in terms of addressing the current agenda, these efforts will pay off in the long run, ensuring standardization and efficiency in your working practices.

We believe that following these outlined principles can substantially aid in establishing new neuroscientific research centers in countries where neuroscience and experimental research have been underrepresented and thus promote implementation of evidence-based approaches in healthcare, and improvement of neuroscientific education and knowledge in a wider community. Finally, in the end this type of pioneering work is about passion. Ignite and follow your passion, for when you are passionate about something, you will find ways to succeed.

## Author Contributions

MI, SM, and OD jointly contributed to the conception of the paper and were involved at different stages in funding acquisition for the Neurolinguistics Laboratory and now the Center for Language and Brain at the HSE University. MI wrote the original draft of the manuscript, that was expanded by SM and OD. All authors contributed to manuscript revision, read, and approved the submitted version.

## Conflict of Interest

The authors declare that the research was conducted in the absence of any commercial or financial relationships that could be construed as a potential conflict of interest.

## Publisher's Note

All claims expressed in this article are solely those of the authors and do not necessarily represent those of their affiliated organizations, or those of the publisher, the editors and the reviewers. Any product that may be evaluated in this article, or claim that may be made by its manufacturer, is not guaranteed or endorsed by the publisher.
